# Consistent temperature dependence of functional response parameters and their use in predicting population abundance

**DOI:** 10.1111/1365-2656.13060

**Published:** 2019-08-09

**Authors:** Louise C. Archer, Esra H. Sohlström, Bruno Gallo, Malte Jochum, Guy Woodward, Rebecca L. Kordas, Björn C. Rall, Eoin J. O’Gorman

**Affiliations:** ^1^ Department of Life Sciences Imperial College London London UK; ^2^ School of Biological, Earth and Environmental Sciences University College Cork Cork Ireland; ^3^ J.F. Blumenbach Institute of Zoology and Anthropology University of Goettingen Goettingen Germany; ^4^ German Centre for Integrative Biodiversity Research (iDiv) Halle‐Jena‐Leipzig Leipzig Germany; ^5^ Institute of Biodiversity Friedrich Schiller University Jena Jena Germany; ^6^ Institute of Plant Sciences University of Bern Bern Switzerland; ^7^ Institute of Biology Leipzig University Leipzig Germany; ^8^ School of Biological Sciences University of Essex Colchester UK

**Keywords:** aquatic, climate change, consumer–resource, freshwater, population dynamics, predator–prey, predictive modelling, trophic interactions

## Abstract

1. Global warming is one of the greatest threats to the persistence of populations: increased metabolic demands should strengthen pairwise species interactions, which could destabilize food webs at the higher organizational levels. Quantifying the temperature dependence of consumer–resource interactions is thus essential for predicting ecological responses to warming.
2. We explored feeding interactions between different predator–prey pairs in controlled‐temperature chambers and in a system of naturally heated streams. We found consistent temperature dependence of attack rates across experimental settings, though the magnitude and activation energy of attack rate were specific to each predator, which varied in mobility and foraging mode.
3. We used these parameters along with metabolic rate measurements to estimate energetic efficiency and population abundance with warming. Energetic efficiency accurately estimated field abundance of a mobile predator that struggled to meet its metabolic demands, but was a poor predictor for a sedentary predator that operated well below its energetic limits. Temperature effects on population abundance may thus be strongly dependent on whether organisms are regulated by their own energy intake or interspecific interactions.
4. Given the widespread use of functional response parameters in ecological modelling, reconciling outcomes from laboratory and field studies increases the confidence and precision with which we can predict warming impacts on natural systems.

## Graphical abstract

The authors assessed whether species’ responses to warming can be reliably estimated from experimental data. They then integrated feeding parameters with energetic modelling to estimate abundance of predators. This allows the authors to predict predator persistence with warming using a mechanistic approach, with responses varying depending on species’ traits and feeding ecology.

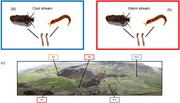

## 1 INTRODUCTION

Global warming is widely predicted to increase the metabolic demands of organisms (Brown, Gillooly, Allen, Savage, & West, 2004; Gilbert et al., 2014), which could strengthen short‐term consumer–resource interactions (O'Connor, 2009; Rall, Vucic‐Pestic, Ehnes, Emmerson, & Brose, 2010) and potentially destabilize ecological communities *via* cascading food web effects (Allesina & Tang, 2012; O'Gorman & Emmerson, 2009). The metabolic theory of ecology (MTE) suggests a predictable exponential scaling of biological rates with temperature, determined by an activation energy of 0.6–0.7 eV (Brown et al., 2004). Contrary to the initial assumptions of MTE, it is now recognized that there is no universal scaling with temperature of metabolic, encounter and attack rates—the key underlying drivers of interaction strength (Clarke, 2006; Dell, Pawar, & Savage, 2011). These rates are also likely shaped by the respective body sizes, foraging strategies and thermal histories of consumers and resources (Dell, Pawar, & Savage, 2014; Sentis, Morisson, & Boukal, 2015). We urgently need to understand the temperature dependence of such interactions and how it varies in different contexts, as this currently limits our ability to predict how warming will affect the higher levels of biological organization beyond single species populations (Uszko, Diehl, Englund, & Amarasekare, 2017).

Characterizing functional responses, which describe per capita feeding rate as a function of resource density (Holling, 1959), and their key parameters of attack rate and handling time can help close this gap. Attack rate describes feeding efficiency and determines consumption at low resource densities (Holling, 1959). Handling time includes the processes of subduing, ingesting and digesting a resource and determines the maximum feeding rate of a consumer (Englund, Öhlund, Hein, & Diehl, 2011). Attack rates typically increase and handling times decrease with warming in functional response experiments, at least up to a thermal optimum of the consumer (Englund et al., 2011; Rall et al., 2012). This could strengthen top‐down control in warmer environments, suppressing the abundance of resource species and potentially even driving them locally extinct (Vasseur & McCann, 2005). Population dynamics of consumers and resources will depend on changes in energy acquisition relative to expenditure, which define an organism's energetic efficiency and its ability to meet its metabolic requirements (Vasseur & McCann, 2005). If warming pushes energetic demands beyond what can be supplied through feeding, consumers could starve and thus decline in population size (Fussmann, Schwarzmüller, Brose, Jousset, & Rall, 2014; Vucic‐Pestic, Ehnes, Rall, & Brose, 2011). Quantifying energetic efficiency may thus be a powerful tool for predicting population abundance in natural systems and indeed whether a population persists or becomes locally extinct.

Since the temperature dependences of functional response parameters influence higher organizational levels, they are increasingly used in models to predict how populations and food webs will change with warming (Binzer, Guill, Rall, & Brose, 2016; Fussmann et al., 2014; Gilbert et al., 2014; Osmond et al., 2017; Petchey, Brose, & Rall, 2010; Vasseur & McCann, 2005). This signals a move beyond climate envelope approaches that ignore biotic interactions and are not mechanistic (Araújo & Luoto, 2007). Given their prominent use in predictive ecological research, it is imperative that we assess the consistency of functional response experiments and their applicability to natural systems. Surprisingly little work has addressed the issue of reproducibility in functional response experiments, for example by repeating experiments, or validating laboratory experiments in the field (Yazdani & Keller, 2016). Furthermore, since the temperature scaling of functional response parameters varies among ecosystems, trophic groups and consumer foraging modes (Dell et al., 2014; Rall et al., 2012), knowledge of how consumers may vary in different experimental settings is also lacking. By testing the temperature dependence of functional response parameters in consumers with contrasting species traits under field conditions, more realistic estimates of feeding interactions can be integrated with energetic requirements to mechanistically explore observed and projected changes in the abundance of natural populations with climate warming.

There are some indications that laboratory experiments may grossly overestimate field‐based feeding rates, although explicit tests are still scarce (Aljetlawi, Sparrevik, & Leonardsson, 2004; Wilhelm, Schindler, & McNaught, 2000). In situ functional response experiments are difficult to carry out and, consequently, they are rare (e.g., Jost, Devulder, Vucetich, Peterson, & Arditi, 2005; Barrios‐O'Neill, Dick, Ricciardi, MacIsaac, & Emmerson, 2014; Novak, Wolf, Coblentz, & Shepard, 2017), so data are often combined from a range of locations with the assumption of spatial and temporal consistency (Angerbjorn, Tannerfeldt, & Erlinge, 1999). Comparative field and laboratory studies are needed to test consistency across contexts (laboratory vs. field) or time (within vs. between years), but the few studies done on this topic are still confounded with the spatial scale of the research (O'Neil, 1997; Wang & Ferro, 1998; Xia, Rabbinge, & Werf, 2003). A more powerful, but rarely implemented approach, due to the challenges of finding suitable study systems, is to combine field‐based assays with natural experiments (e.g., Dunne, Saleska, Fischer, & Harte, 2004; O'Gorman et al., 2014), where thermal gradients can be exploited in situ in the absence of other confounding factors.

To link laboratory experiments, field data and energetic modelling, we sought to determine whether the temperature dependences of laboratory‐derived functional response parameters are (1) repeatable at different times and (2) realistic (i.e., reflecting field conditions). We then tested the utility of these parameters for (3) predicting changes in population abundance and persistence of two consumers with contrasting species traits in response to increasing temperature, highlighting how they may be embedded in the mechanistic and predictive study of biotic responses to warming.

## 2 MATERIALS AND METHODS

### 2.1 Study site

**Figure 1:**
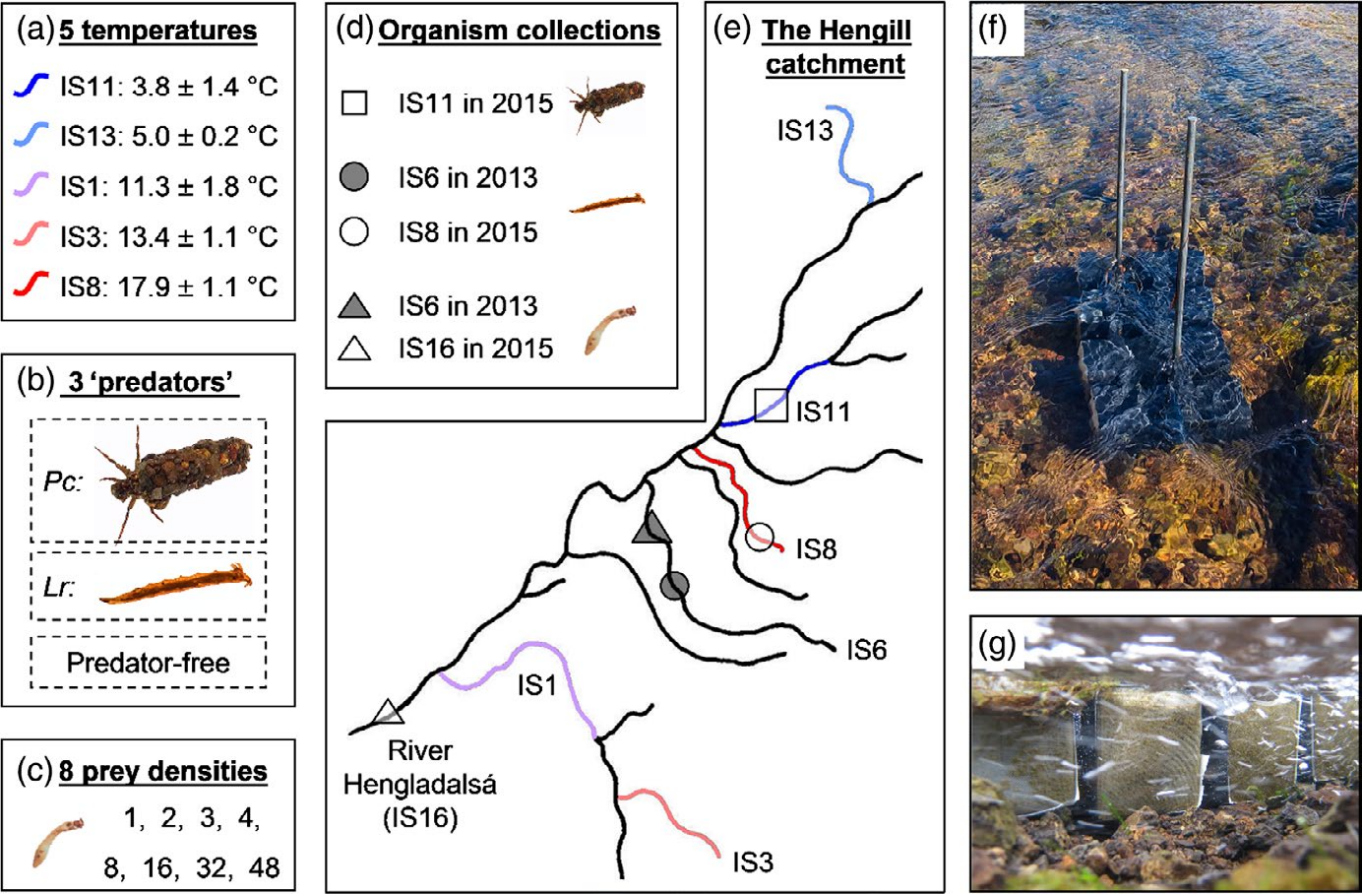
Overview of the field experimental design: (a) five stream temperatures (mean ± *SD*); (b) three predator treatments, including the cased caddisfly larva *Potamophylax cingulatus* (*Pc*), the housefly larva *Limnophora riparia* (*Lr*) and a predator‐free control; and (c) eight prey densities of the blackfly larvae (Simuliidae). (d) The stream each taxon was collected from in each year of the experiments. (e) Map of the Hengill geothermal stream catchment, highlighting the streams where the experimental microcosms were deployed for the field experiments, with colours and labels corresponding to (a), and where the organisms used in the experiments were collected, with symbols corresponding to (d). Note that the mean temperatures of IS6 and IS16 during organism collections were 17.4°C and 7.5°C, respectively. Microcosms in situ in (f) plane and (g) side elevation

**Figure 2:**
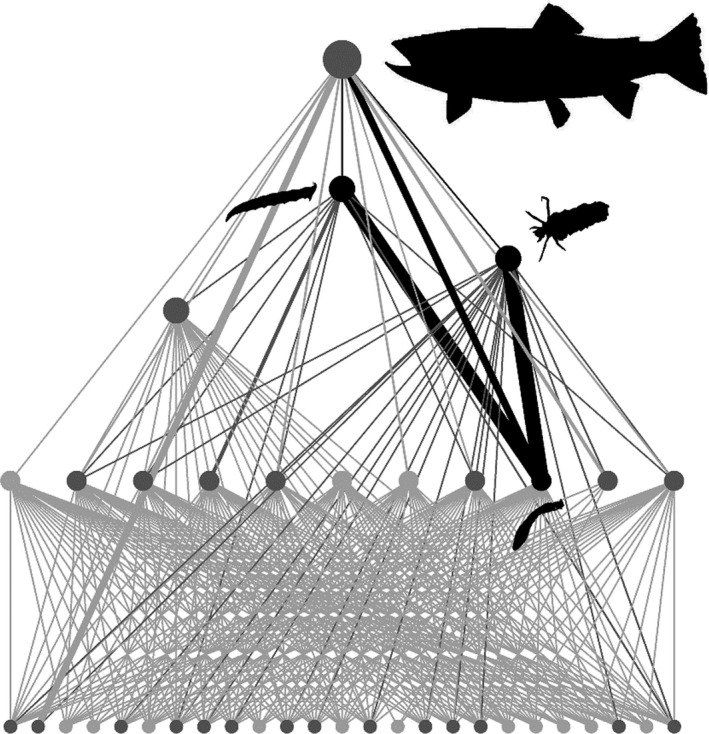
Food web for a stream in the Hengill valley, where circles are species, lines are feeding interactions (described in O’Gorman et al., 2019) and the thickness of the lines is the relative biomass of each resource species in that predator's diet. Black circles and lines correspond to the organisms used in this study and predatory links to them. Dark grey circles and lines correspond to organisms involved in feeding interactions with the organisms in this study, while light grey circles and lines are other species in the food web that do not have direct feeding interactions with them. The key macroinvertebrate predators in the system are *Limnophora riparia* and *Potamophylax cingulatus*, who exert strong feeding pressure on the abundant, filter‐feeding Simuliidae (cf. Figure 1 for definitions of silhouettes). At the top of the food web, brown trout exert weak top‐down control on *L. riparia* and *P. cingulatus*, with their strongest links to Simuliidae and adult Diptera

We conducted in situ functional response experiments across a natural temperature gradient by using geothermally heated streams in Hengill, southwest Iceland (Friberg et al., 2009; Woodward et al., 2010; O'Gorman et al., 2016; Figure 1a,e). The system includes the river Hengladalsá and 15 of its tributaries (Demars et al., 2011), which range in temperature from about 4 to 25°C, but are otherwise physically and chemically similar (Demars et al., 2011; Friberg et al., 2009). This enables us to isolate the in situ effects of temperature on the constituent organisms without other confounding environmental effects (O'Gorman et al., 2014). Previous work on the Hengill system has identified the major predatory invertebrate taxa as the dipteran larva *Limnophora riparia* (Fallén) and the caddisfly larva *Potamophylax cingulatus* (Stephens). Both species exert strong feeding pressure on abundant blackfly larvae from the Simuliidae family and experience weak top‐down control from their only predator, brown trout, *Salmo trutta* (Figure 2). We carried out experiments involving *L. riparia*, *P. cingulatus* and Simuliidae as key trophic motifs determining energy flow through the Hengill streams. Initial laboratory work was performed at the University of Iceland in July 2013; all subsequent laboratory and field experiments were carried out in May–June 2015.

### 2.2 Study organisms

Third‐instar larvae of *L. riparia* and fifth‐instar larvae of *P. cingulatus* were used as predators in the field and laboratory experiments. *Limnophora riparia* is a true fly from the Muscidae family, widely distributed in the Palaearctic region (Skidmore, 1985) and common in fast‐flowing streams (Wotton & Merritt, 1988). It is an active suctorial predator during its third larval instar, with a preferred diet of larval and pupal Simuliidae (Merritt & Wotton, 1988; Werner & Pont, 2003). Larvae of *L. riparia* (7.9 ± 0.2 mm in 2013; 10.4 ± 1.4 mm in 2015; mean body length ± *SD*) were collected from streams where they were most abundant across study periods (Figure 1b,d). *Potamophylax cingulatus* is a cased caddisfly from the Limnephilidae family, widely distributed in European streams, particularly at higher latitudes and elevations (Gíslason, Hannesdóttir, Munoz, & Pálsson, 2015). It is an active, benthic forager, which is typically considered a shredder of leaf litter in stream ecosystems (Otto, 1974), but becomes predatory in the fourth and fifth instars (Giller & Sangpradub, 1993). Larvae of *P. cingulatus* (1.9 ± 0.3 mm; mean head capsule width ± *SD*) were collected from the stream where they were most abundant in 2015 (Figure 1b,d).

The prey species for both predators were blackfly larvae from the Simuliidae family, including *Simulium aureum* (Fries), *S. vernum* (Macquart), *S. vittatum* (Zetterstedt) and *Prosimulium ursinum* (Edwards). Simuliidae larvae are largely sedentary filter feeders, typically associated with fast‐flowing waters (Wallace & Merritt, 1980). They commonly comprise a large proportion of benthic biomass (Cummins & Klug, 1979; Werner & Pont, 2003), making them an important food resource for many freshwater predators (Malmqvist, Adler, Kuusela, Merritt, & Wotton, 2004; O'Gorman et al., 2016). The species used in this study are all similarly sized, functionally equivalent and difficult to tell apart in the field, so were treated as a single prey type. Simuliidae larvae (5.8 ± 1.2 mm in 2013; 7.1 ± 1.4 mm in 2015; mean body length ± *SD*) were collected from the same stream as the predator in 2013 and from the river Hengladalsá in 2015, given that predator species from two different streams were used in that year (Figure 1c,d).

### 2.3 Population abundance

We used data collected from the Hengill streams over an 8‐year period to explore the intergenerational effect of temperature on the population abundance of *L. riparia*, *P. cingulatus* and their Simuliidae prey. Macroinvertebrates were quantitatively Surber‐sampled from 14 streams in the Hengill catchment in August 2004, 2008 and 2012 (25 × 20 cm quadrat; 200 μm mesh; *n* = 5 per stream per year). The samples were preserved in 70% ethanol, and the number of *L. riparia*, *P. cingulatus* and Simuliidae individuals found in each was recorded. An average abundance was estimated from the five Surber samples (since they are not independent replicates) for a single estimate of abundance for each species per stream per year. The effect of temperature on population abundance was analysed with a generalized additive mixed‐effects model (GAMM), with year as a random effect to account for potential temporal autocorrelation in population abundances (*gamm* function in the mgcv package of R). All statistical analyses were performed in R 3.5.0.

### 2.4 Laboratory experiments

To assess the repeatability of functional response experiments, laboratory trials were carried out in 2013 and 2015 in the same controlled‐temperature (CT) chamber (GRAM K400LE, type 3011‐1F4B) at the University of Iceland. We will refer to these studies as Lab 2013 and Lab 2015 henceforth. *Limnophora riparia* was used as a predator in both years, while *P. cingulatus* was included as a comparative predator species in the Lab 2015 study. Organisms were collected in the streams at Hengill and immediately transported to CT chambers, where they were stored in aquaria filled with water from the river Hengladalsá, continuously aerated using air pumps and maintained at temperatures that matched their respective natal streams (Figure 1).

Plastic cylindrical microcosms (7.3 cm diameter, 11.5 cm height) filled with air‐saturated water collected from the river Hengladalsá (50 ml in 2013 and 100 ml in 2015) served as experimental arenas. Each experimental unit held one predator individual and one of eight initial densities of prey (Figure 1c). A predator‐free control was added for every prey density in each experimental trial to assess natural prey mortality. Predators were starved for 24 hr prior to the beginning of each experiment to allow sufficient time for gut evacuation (Vucic‐Pestic et al., 2011). All prey individuals were placed in the arenas 30 min before the predators were added to allow them to acclimatize. Experimental trials were run at four temperatures (5, 10, 15 and 18°C in 2013; and 4, 6, 10 and 18°C in 2015) for 24 hr. At the end of this experimental period, predators were removed, and the surviving prey were counted. Experimental units where the predator had died or pupated were discounted because they could no longer feed (9 out of 168 and 8 out of 214 experimental units were discounted for *L. riparia* and *P. cingulatus*, respectively). Pupated prey were still counted if they were alive, however, because they are still vulnerable to predation (Wotton & Merritt, 1988).

### 2.5 Field experiments

Field experiments were carried out in 2015 in five Hengill streams that differed in temperature (Figure 1a,e). We refer to this study as Field 2015 henceforth. Black Perspex cuboidal microcosms (15 × 8 × 8 cm) sealed with 250‐μm nylon mesh were used as experimental arenas placed in situ in the streams, which allowed for approximation of natural stream conditions (i.e., exposed to natural variation in stream flow, turbidity, dissolved oxygen and nutrients). Microcosms were assembled into blocks of four and anchored to the benthos in each stream, perpendicular to the flow (Figure 1f,g). All four microcosms in each block had one of eight initial densities of prey (Figure 1c), with three microcosms containing one predator (either *L. riparia* or *P. cingulatus*) and the fourth being a predator‐free control. Predators were starved for 24 hr prior to the start of an experiment by placing them in otherwise empty microcosms anchored to the benthos of their natal stream. All prey individuals were collected on the same day as the field experiments and, as in the laboratory experiments, were placed in the arenas for 30 min before adding the predators. Experiments ran for 24 hr, after which time predators were removed from each arena and the number of surviving prey was recorded. Experimental units were again discounted if the predator had died or pupated (2 out of 78 and 1 out of 79 experimental units discounted for *L. riparia* and *P. cingulatus*, respectively).

### 2.6 Quantifying feeding rate

To account for natural prey mortality, we numerically integrated prey decline in the predator‐free controls over the experimental duration (*t* = 1 day), as per Rosenbaum and Rall (2018), using the following equation:

(1) $$\frac{\text{d}N_{\mathrm{ij}}}{\text{d}t}=-m_{\mathrm{ij}}N_{\mathrm{ij}}$$

where *m_ij_* is the natural mortality rate (individuals/day), *N_ij_* is the initial number of prey (individuals/arena), the subscript *i* refers to the Lab 2013, Lab 2015 or Field 2015 data, and the subscript *j* refers to either *L. riparia* or *P. cingulatus*. Temperature dependence was incorporated into Equation 1 according to MTE (Brown et al., 2004; Rall et al., 2012), by scaling *m_ij_* by an Arrhenius temperature term:

(2) $$m_{\mathrm{ij}}=m_{ij0}e^{E_{m_{\mathrm{ij}}}\frac{T_{i}-T_{0}}{kT_{i}T_{0}}}$$

where *m_ij_*_0_ is natural mortality at *T*_0_, *E_mij_* is the activation energy (eV), *k* is the Boltzmann constant (8.618 × 10^–5^ eV/K), *T_i_* is the absolute experimental temperature (K) and *T*_0_ is 283.15 K (i.e., 10°C, the mid‐point of the range of temperatures used across all experiments).

We used the R package odeintr to solve the ordinary differential equation for natural mortality, as described in Rosenbaum and Rall (2018). The point estimates for the free parameters in the model were obtained by maximum likelihood using the '*mle2*' function with '*method = "L‐BFGS‐B"*' from the R package bbmle. The optimum values of *m_ij_*_0_ and *E_mij_* for the Lab 2015 and Field 2015 experiments of both predator–prey combinations were taken from the most parsimonious model according to Bayesian information criterion (BIC) and used to correct for natural mortality in all subsequent analyses (Table S1; Figures S1 and S2). The inclusion of the Lab 2013 data (where there was no natural mortality and so *m_ij_*_0_ = 0) allowed us to determine whether any temperature‐dependent feeding patterns were consistent after correcting for natural mortality in the Lab 2015 and Field 2015 datasets.

The functional response describes the per capita feeding rate, *F* (individuals/day), of a predator in dependence of prey density, *N*:

(3) $$F=\frac{\mathrm{aN}}{1+abN}$$

where *a* is the attack rate (m^2^/day) and *b* is the handling time (days/individual). The attack rate can be further described by:

(4) $$a=cN^{h-1}$$

where *c* is the attack coefficient, describing the linear increase in attack rate, and *h* is the Hill exponent, which determines the shape of the functional response. Classically, the functional response has been categorized into linear type I (*h* = 1 and *b* = 0, with a cut‐off for maximum feeding rate), hyperbolic type II (*h* = 1) and sigmoidal type III (*h* = 2). Thus, the change in prey density through time without replacement (including a correction for natural mortality, where appropriate) can be described as:

(5) $$\frac{\text{d}N_{\mathrm{ij}}}{\text{d}t}=\frac{-c_{\mathrm{ij}}N_{\mathrm{ij}}^{h_{\mathrm{ij}}}}{1+c_{\mathrm{ij}}b_{\mathrm{ij}}N_{\mathrm{ij}}^{h_{\mathrm{ij}}}}-m_{\mathrm{ij}}N_{\mathrm{ij}}$$

The temperature dependences of *b_ij_* and *c_ij_* were incorporated into Equation 5 by scaling each of these parameters by an Arrhenius temperature term, as for *m_ij_* in Equation 2:

(6) $$b_{\mathrm{ij}}=b_{ij0}e^{E_{b_{\mathrm{ij}}}\frac{T_{i}-T_{0}}{kT_{i}T_{0}}}$$

(7) $$c_{\mathrm{ij}}=c_{ij0}e^{E_{c_{\mathrm{ij}}}\frac{T_{i}-T_{0}}{kT_{i}T_{0}}}$$

We fitted type I, II and III responses separately; thus, each of our functional response models included four free parameters for each dataset (*b_ij_*_0_, *E_bij_*, *c_ij_*_0_ and *E_cij_*). All possible combinations of temperature dependence across settings were fitted by sequentially letting *E_bij_* = 0 and *E_cij_* = 0 and replacing *E_bij_* with *E_bj_*, *b_ij_*_0_ with *b_j_*_0_, *E_cij_* with *E_cj_* and *c_ij_*_0_ with *c_j_*_0_ (i.e., a single *E_bj_, b_j_*_0_, *E_cj_* or *c_j_*_0_ for all datasets combined), resulting in 55 different models for each predator species (Tables S2 and S3). Equation 5 was solved using the same model fitting procedure as described above for determining natural mortality. Model fittings were compared using BIC, where the lowest BIC value determines the most parsimonious model, which was subsequently used to calculate prey consumption rates (Tables S2 and S3).

### 2.7 Quantifying metabolic rate

To estimate the energetic requirements of consumers, we first determined the temperature dependence of routine metabolic rate (sensu Ikeda 2016) by measuring the oxygen consumption rate of individual *L. riparia* and *P. cingulatus* at 5, 10, 15, 20 and 25°C, according to Brodersen, Pedersen, Walker, and Jensen (2008). Individuals were collected from the same streams used in the 2015 experiments and immediately transported to the laboratory, where they were stored in aquaria within CT chambers (as described above) for approximately 24 hr prior to measurements. This ensured that animals could clear their guts prior to experiments, since digestion can affect metabolic measurements. Before each oxygen consumption experiment, individuals were confined in glass chambers and acclimatized to the experimental temperature for 15 min. The glass chambers were completely filled (i.e., no headspace) with water from the river Hengladalsá, which was filtered through a 0.45‐µm Whatman membrane filter and bubbled to reach 100% oxygen saturation. A magnetic stir bar was placed at the bottom of each chamber but separated from the organism by a mesh screen. In each trial, one individual *L. riparia* or *P. cingulatus* was placed in each of seven chambers and the eighth chamber was used as an animal‐free control to account for sensor drift.

Oxygen consumption was measured with an oxygen microelectrode (MicroResp; Unisense, Denmark) fitted through a capillary in the gas‐tight stopper of each chamber. Three measurement periods were recorded for each individual predator (10–15 s each, where oxygen concentration was measured every second). Metabolic rates (µmol O_2_/hr) were calculated as the best‐fitting line through all data points measured in each chamber, corrected for background rates in the animal‐free control chamber and then converted to energetic equivalents (J/h) using atomic weight (1 mol O_2_ = 31.9988 g), density (1.429 g/L) and a standard conversion (1 ml O_2_ = 20.1 J; Peters, 1983). Metabolic rate was measured for 5–10 individuals of each species at each experimental temperature, with a new individual used in every trial. The body length of *L. riparia* and head width of *P. cingulatus* were measured after each trial to estimate individual dry mass from length–weight relationships established for the system for *L. riparia* (Figure S3) and from the literature for *P. cingulatus* (Meyer, 1989).

Metabolic rate, *I* (J/h), depends on both temperature and body mass according to MTE (Brown et al., 2004):

(8) $$I_{j}=I_{j0}M_{j}^{d_{\mathrm{Ij}}}e^{E_{\mathrm{Ij}}\frac{T_{I}-T_{0}}{kT_{I}T_{0}}}$$

where *I_j_*_0_ is metabolic rate at *T*_0_, *d_Ij_* is an allometric exponent, *M_j_* is dry body mass (mg) and *E_Ij_* is the activation energy (eV). We performed a multiple linear regression on the natural logarithm of Equation 8, exploring all possible combinations of the main and interactive effects of temperature and body mass on metabolic rate. We also explored a quadratic term for temperature to account for potential curvature in metabolic rates at higher temperatures. The model with the lowest BIC value for each predator was chosen as the best‐fitting model (Table S4).

### 2.8 Estimating energetic efficiency

The ratio of feeding to metabolism (i.e., energetic efficiency) of *L. riparia* and *P. cingulatus* was calculated according to Rall et al. (2010) and Vucic‐Pestic et al. (2011). First, the per capita energy feeding rate, *F_Ejk_* (J/h), was estimated as:

(9) $$F_{\mathrm{Ejk}}=F_{\mathrm{jk}}M_{S}E_{S}$$

where *F_jk_* is the feeding rate of predator *j* in stream *k*, *M_S_* is the ash‐free dry body mass (AFDM) of Simuliidae individuals and *E_S_* is the energy content of Simuliidae. We estimated *F_jk_* by parameterizing Equations (5), (6), (7) with *c_ij_* and *b_ij_* from the best‐fitting functional response model for each species (where *i* = Field 2015), *N_ij_* as the average abundance of Simuliidae (across August 2004, 2008 and 2012) for each of 14 streams in the Hengill system, and *T*_i_ as the mean temperature of each stream across August 2004, 2008 and 2012 (see the 'Population abundance' section above). We estimated *M_S_* = 0.546 mg (AFDM) from the mean length of Simuliidae in the Field 2015 experiments and an established length–weight relationship (Benke, Huryn, Smock, & Wallace, 1999), and we estimated *E_S_* = 23.1 J/mg (after Cummins, 1967).

The assimilation efficiency, *ω*, determines the fraction of energy ingested by animals that is not lost to excretion and can be described as the ratio (from 0 to 1) of assimilated energy to consumed energy (Lang, Ehnes, Brose, & Rall, 2017). While *ω* is traditionally assumed to be a constant for carnivores in population modelling (*ω* = 0.85; after Yodzis & Innes, 1992), a recent meta‐analysis showed that it scales systematically with temperature as:

(10) $$\omega_{k}=\frac{\omega_{0}e^{E_{\omega_{k}}\frac{T_{k}-T_{0_{*}}}{kT_{k}T_{0_{*}}}}}{1+\omega_{0}e^{E_{\omega_{k}}\frac{T_{k}-T_{0_{*}}}{kT_{k}T_{0_{*}}}}}$$

where *ω_0_* = *e* ^2.266^ is the intercept of the linearized version of Equation 10 at T_0__*_ = 293.15 K (see Lang et al., 2017), *E_ωk_* = 0.164 eV is the activation energy for carnivorous invertebrates (after Lang et al., 2017) and we set *T_k_* as the mean temperature of each stream *k* across August 2004, 2008 and 2012.

The dimensionless energetic efficiency, *y_jk_*, was then calculated as:

(11) $$y_{\mathrm{jk}}=\frac{\omega_{k}F_{\mathrm{Ejk}}}{I_{j}}$$

For example, if *y_jk_* = 2, this suggests that the feeding rate of predator *j* is twice its metabolic demand in stream *k*, and if *y_jk_* < 1, this suggests that the feeding rate of predator *j* is insufficient to meet its metabolic demand in stream *k*. While metabolic rates are sometimes converted from basal into field rates according to a constant (e.g., a factor of 3; after Savage et al., 2004), predators were observed to be active within respirometry chambers, and so the measured rates were used as an approximation of field metabolic rates.

We fitted generalized additive models (GAM) to estimate the relationship between energetic efficiency, *y_jk_*, and temperature, *T_k_* (*gam* function in the mgcv package of R). We also used GAM to determine whether energetic efficiencies can be used to predict the population abundance of consumer species, with *y_jk_* as the dependent variable and *N_jk_* as the independent variable, where *N_jk_* is the average population abundance of predator *j* in stream *k* across August 2004, 2008 and 2012.

## 3 RESULTS

### 3.1 Population abundance

**Figure 3:**
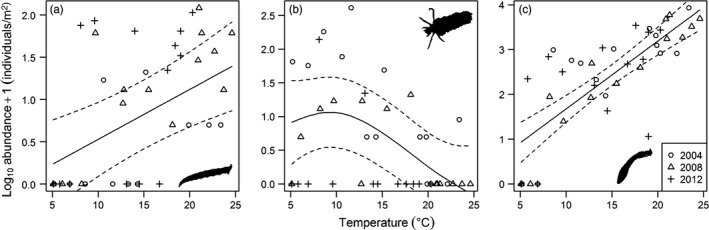
Long‐term trends in the population abundances of (a) *Limnophora riparia*, (b) *Potamophylax cingulatus* and (c) Simuliidae in 14 streams of different temperature in the Hengill catchment. Abundance was quantified in the month of August in different years: 2004 (circles), 2008 (triangles) and 2012 (crosses). Solid lines are the significant relationships between temperature and log_10_ abundance from generalized additive mixed‐effects models, and dashed lines are the 95% confidence intervals of model fittings

*Limnophora riparia* was absent from the coldest streams in the system, and its population abundance increased with increasing temperature (GAMM: *F* = 12.36, *p* = .001; *r* ^2^ = .17; Figure 3a). The population abundance of *P. cingulatus* exhibited a hump‐shaped response to temperature, increasing to a peak around 10°C, with the species rarely found in the warmest streams (GAMM: *F* = 8.97, *p* < .001; *r* ^2^ = .21; Figure 3b). Simuliidae were absent from the coldest streams, and their population abundance increased with temperature to a maximum of 8,500 individuals/m^2^ (GAMM: *F* = 58.58, *p* < .001; *r* ^2^ = .58; Figure 3c).

### 3.2 Feeding rate

**Figure 4:**
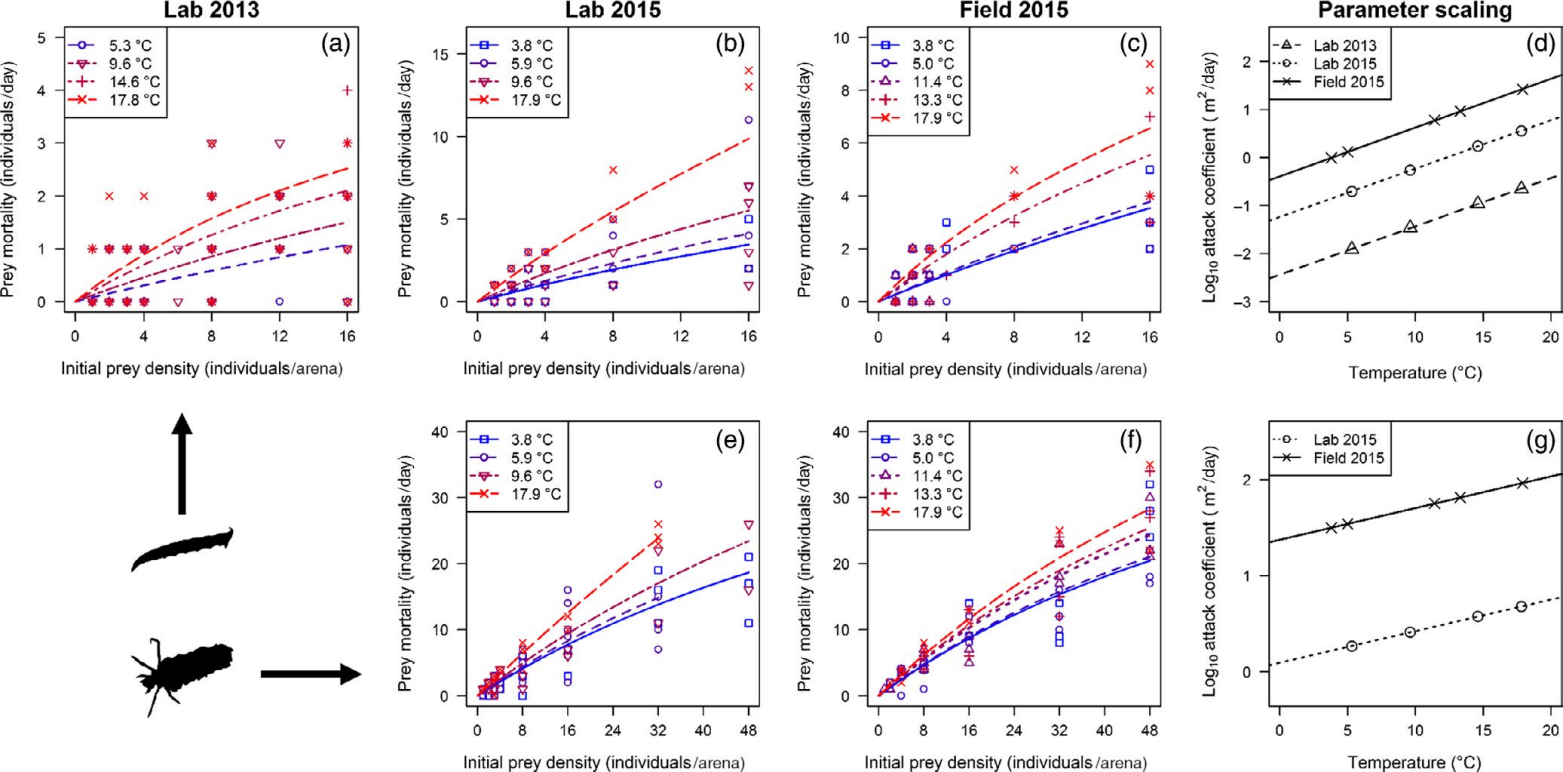
Functional response of (a–d) *Limnophora riparia* and (e–g) *Potamophylax cingulatus* feeding on Simuliidae across a gradient of temperatures. Experiments were conducted in (a) the laboratory in 2013, (b,e) the laboratory in 2015 and (c,f) the field in 2015. The consistent temperature dependence of feeding at low prey density (i.e., the attack coefficient) is also shown for (d) *L. riparia* and (g) *P. cingulatus*, using parameters estimated from the most parsimonious model according to BIC (Table 1)

The combined Lab 2013, Lab 2015 and Field 2015 datasets for *L. riparia* feeding on Simuliidae were best described by a type II functional response with separate intercept values of attack coefficient for each dataset and a shared handling time (Table 1; Figure 4a–c). The model included a shared activation energy of attack coefficient of 0.70 ± 0.30 eV (mean ± 95% CI), indicating a similar temperature dependence of attack rate in all three sets of experiments, while handling time was independent of temperature (Table 1). Thus, the attack coefficient increased significantly with temperature, independent of year or experimental setting (Figure 4d), and the shared handling time indicated that maximum feeding rate was similar between the Lab 2013, Lab 2015 and Field 2015 datasets (Figure S4a–c). This result addressed objectives 1 and 2 of the study by demonstrating a consistent temperature dependence of the functional response between years and across settings.

**Table 1:** Parameter estimates with associated standard errors (*SE*), *z*‐values and *p*‐values for the most parsimonious models according to Bayesian information criterion (Tables S2 and S3) describing the functional response of *Limnophora riparia* and *Potamophylax cingulatus*. Parameters correspond to those listed in Equations 6 and 7, where *c*_0_ is attack coefficient at *T*_0_, *b*_0_ is handling time at *T*_0_ and *E_c_* is the activation energy of attack coefficient. For each predator, the best‐fitting model was a type II functional response with separate estimates of *c*_0_ for the Lab 2013, Lab 2015 and Field 2015 data

Species	Parameter	Dataset	Estimate	*SE*	*z*‐value	*p*‐value
*L. riparia*	*c*_0_	Lab 2013	0.241	.171	−8.325	<.001
	*c*_0_	Lab 2015	0.802	.218	−1.010	.313
	*c*_0_	Field 2015	1.889	.281	2.260	.024
	*b*_0_	Combined	4.033	.394	3.543	<.001
	*E_c_*	Combined	0.704	.152	4.615	<.001
*P. cingulatus*	*c*_0_	Lab 2015	1.529	.088	4.849	<.001
	*c*_0_	Field 2015	5.515	.103	16.61	<.001
	*b*_0_	Combined	0.644	.160	−2.756	.006
	*E_c_*	Combined	0.229	.067	3.424	<.001

The combined Lab 2015 and Field 2015 datasets for *P. cingulatus* feeding on Simuliidae were best described by a type II functional response with separate intercept values of attack coefficient for each dataset and a shared handling time (Table 1; Figure 4e–f). The model included a shared activation energy of attack coefficient of 0.23 ± 0.13 eV (mean ± 95% CI), indicating a similar temperature dependence of attack rate in both sets of experiments, while handling time was again independent of temperature (Table 1). Thus, as for *L. riparia*, the attack coefficient increased significantly with warming, independent of experimental setting (Figure 4g), and the shared handling time indicated that maximum feeding rate was similar in the Lab 2015 and Field 2015 datasets (Figure S4d–e). This result further supports our finding of a consistent temperature dependence of the functional response across experimental settings (objective 2).

### 3.3 Metabolic rate

**Figure 5:**
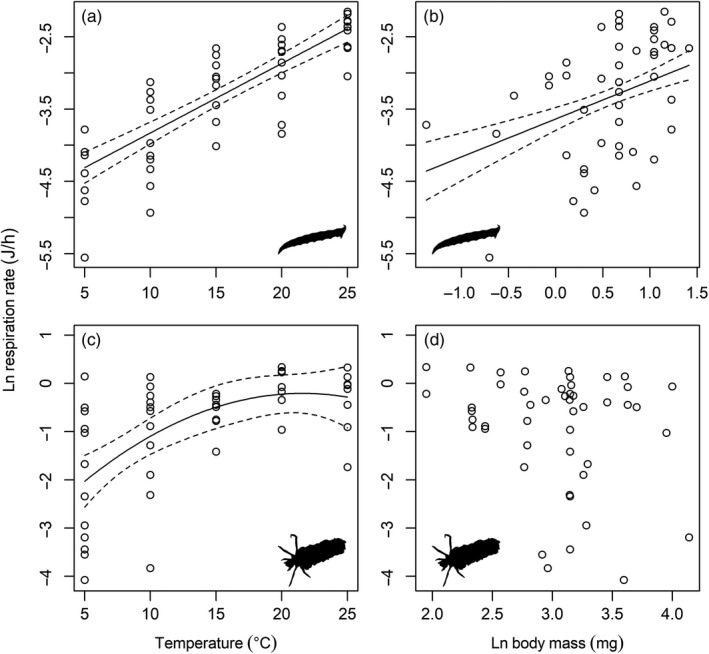
Body mass and temperature dependence of respiration rate for the two predators. The respiration rate of *Limnophora riparia* was best described by a loglinear model including the significant effect of (a) temperature and (b) body mass (Table 2). (c) The respiration rate of *Potamophylax cingulatus* was best described by a polynomial model including the significant effect of temperature only (Table 2). (d) There was no significant relationship between respiration rate and body mass in *P. cingulatus*. Note that the lines of best fit for the explanatory variables in panels (a) and (b) are shown after setting the other explanatory variable to its mean value

The respiration rate of *L. riparia* was best described by a multiple linear regression model that included the significant main effects of temperature and body mass (*F*_2,43_ = 90.28; *p* < .001; *r* ^2^ = .80; Table 2). The respiration rate of *L. riparia* increased with temperature with an activation energy of 0.69 ± 0.12 eV (mean ± 95% CI; Figure 5a) and with body mass with an allometric exponent of 0.53 ± 0.20 (mean ± 95% CI; Figure 5b). The respiration rate of *P. cingulatus* was best described by a polynomial regression model that included the significant main linear and quadratic effects of temperature only (*F*_1,38_ = 19.95; *p* < .001; *r* ^2^ = .34; Table 2). The respiration rate of *P. cingulatus* increased with temperature up to 21.5°C with an activation energy of 1.07 ± 0.51 eV (mean ± 95% CI; Figure 5c), and there was no effect of body mass (Figure 5d).

**Table 2:** Parameter estimates with associated standard errors (*SE*), *t*‐values and *p*‐values for the most parsimonious model according to Bayesian information criterion (Table S4) describing the respiration rates of *Limnophora riparia* and *Potamophylax cingulatus*. Parameters correspond to those listed in Equation 8, where *I*_0_ is metabolic rate at *T*_0_, *d_I_* is the allometric exponent and *E_I_* is the activation energy. Note the addition of a quadratic temperature term ($E_{I}^{2}$) in the best‐fitting model for *Potamophylax cingulatus*

Species	Parameter	Estimate	*SE*	*t*‐value	*p*‐value
*L. riparia*	*I*_0_	−4.171	.089	−47.10	<.001
	*d_I_*	0.525	.099	5.296	<.001
	*E_I_*	0.687	.059	11.72	<.001
*P. cingulatus*	*I*_0_	−1.056	.191	−5.540	<.001
	*E_I_*	1.072	.254	4.219	<.001
	$E_{I}^{2}$	−0.339	.172	−1.974	.054

### 3.4 Energetic efficiency

**Figure 6:**
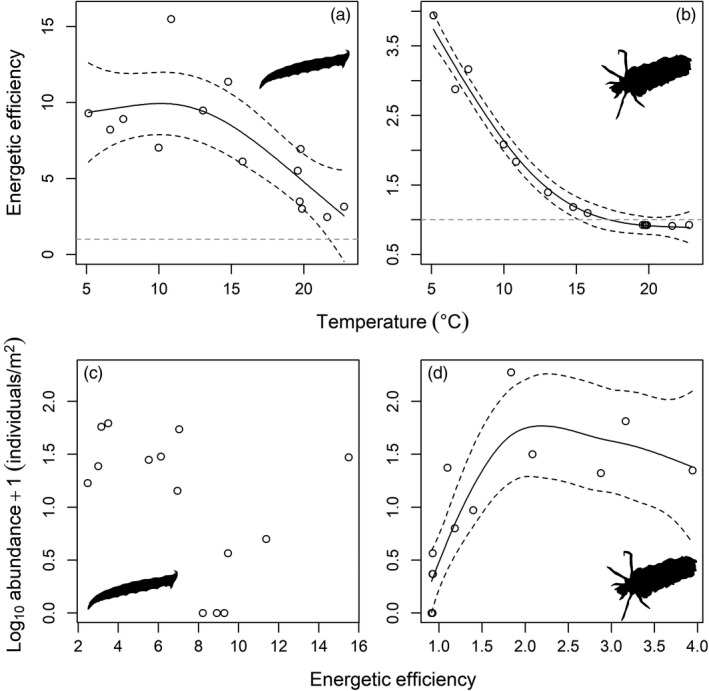
Relationships between energetic efficiency and temperature for (a) *Limnophora riparia* and (b) *Potamophylax cingulatus* feeding on natural densities of Simuliidae from 14 streams of different temperature in the Hengill catchment. Data points above and below the dashed grey line indicate scenarios where energy intake is greater than (*y* > 1) and less than (*y* < 1) energy expenditure, respectively. Energetic efficiency was a poor predictor of population abundance in (c) *L. riparia*, but it was an excellent predictor of population abundance in (d) *P. cingulatus*. Solid lines are the significant relationships from generalized additive models, and dashed lines are the 95% confidence intervals of model fittings

Energetic efficiency declined as temperature increased for both *L. riparia* (GAM: *F* = 7.06, *p* = .009; *r* ^2^ = .56; Figure 6a) and *P. cingulatus* (GAM: *F* = 147.6, *p* < .001; *r* ^2^ = .98; Figure 6b). *Limnophora riparia* was energetically efficient across all temperatures in the sampled streams, with a minimum energetic efficiency of 2.5 (Figure 6a). There was no significant relationship between energetic efficiency and population abundance in *L. riparia* (GAM: *F* = 4.09; *p* = .067; Figure 6c). Energetic efficiency was <1 in the warmest streams for *P. cingulatus*, indicating that the species was energetically inefficient above 17°C (Figure 6b). There was a significant increase in population abundance as energetic efficiency increased for *P. cingulatus* (GAM: *F* = 17.69; *p* < .001; *r* ^2^ = .72; Figure 6d). This result partially supports the utility of functional response parameters for predicting changes in population abundance (objective 3).

## 4 DISCUSSION

### 4.1 Feeding rate

The attack rate of both predators increased exponentially with temperature, but only the activation energy for *L. riparia* was in line with a meta‐analysis of functional response experiments (Rall et al., 2012). The temperature dependence of attack rate was much weaker for *P. cingulatus*, highlighting the potential importance of species traits for quantitatively predicting the temperature dependence of feeding rates in different consumers (Dell et al., 2014). *Potamophylax cingulatus* is much faster than, and thus more likely to encounter, its relatively sedentary Simuliidae prey, making it energetically profitable for it to feed even at low prey densities (Dell et al., 2014; Sentis, Hemptinne, & Brodeur, 2012). In contrast, *L. riparia* is a slower‐moving predator with a suctorial feeding mechanism, which makes capturing its prey a lengthy process (Wotton & Merritt, 1988). This is likely to increase handling time and reduce its overall attack rate. Although hump‐shaped relationships between attack rate and temperature have been previously demonstrated (Englund et al., 2011), such a response was not indicated here, suggesting that neither predator was physiologically limited by the range of temperatures experienced in the laboratory or field (i.e., 3–18°C).

The consistent temperature scaling of attack rate across experimental settings suggests that laboratory functional response experiments could be an accurate and repeatable predictor of field consumption rates under scenarios of warming. This has rarely been tested in a systematic way (O'Neil, 1997; Wang & Ferro, 1998; Xia et al., 2003) and offers great promise for their use in predictive ecological research. Nevertheless, our field experiments should be interpreted only as an approximation of natural systems, and the consistency between laboratory and field may have been driven by the use of similar artificial containers in both settings, requiring further validation, for example, with observational approaches that are immensely difficult to implement for freshwater invertebrates. Recent advances with in situ video‐tracking technology may help to overcome this difficulty (Cloyed, Dell, Hayes, Kordas, & O’Gorman, 2019).

Natural mortality of the prey occurred throughout the 2015 experiments, though not in 2013, which may have been related to the different seasons in which the experiments were run, or interannual differences in the condition of the prey. This warrants some caution when extrapolating the results to field‐based consumption rates; however, the consistent temperature effect on feeding rates from experiments with and without natural mortality suggests that our statistical correction for this unexpected loss of prey was effective. In addition, the intercepts of attack rate differed between experimental settings, suggesting a degree of contingency in the absolute magnitude of this parameter. This was most likely associated with small differences in the structure and dimensions of the laboratory and field arenas. As a result, while the temperature dependence of the functional response may be well characterized, some caution should be exercised when extrapolating absolute measures in the wild from experimental data.

While the activation energy of attack rate was consistent across experimental settings for each predator species, there was no effect of temperature on handling time, which is in contrast to many previous studies (Rall et al., 2012; Sentis et al., 2012, 2015; Vucic‐Pestic et al., 2011). Handling time is also constrained by morphological and behavioural determinants such as predator feeding apparatus, gut capacity and prey armature and thus may be quantitatively less affected by temperature than attack rate (Giller, 1980; Woodward & Hildrew, 2002). For example, prey defence (hard vs. soft integument) has previously been shown to be a more important determinant of handling time in predator–prey interactions than temperature (Kalinoski & DeLong, 2016). Indeed, feeding rates of both *L. riparia* and *P. cingulatus* were well described by a single handling time across settings, suggesting a similar maximum feeding rate (within predators) in all experiments. Given that consumer–resource interactions are strongly dependent on body mass (Rall et al., 2012; Schneider, Scheu, & Brose, 2012), the lower handling time and thus greater maximum feeding rate in *P. cingulatus* were likely driven by its larger predator–prey body mass ratio compared to *L. riparia*. Here, *P. cingulatus* must consume many prey (which are small relative to its own size) while the suctorial feeding mode of *L. riparia* (Merritt & Wotton, 1988) means it can consume fewer, relatively large prey to meet its metabolic demands. This underscores the importance of species traits in determining the magnitude of responses to warming.

### 4.2 Metabolic rate

The activation energy of metabolic rate was consistent with MTE, with 95% CI for both predators including the expected range of 0.6–0.7 eV (based on the average of observed metabolic rates; Brown et al., 2004). *Limnophora riparia* did not appear to be physiologically limited over the 5–25°C range of experimental temperatures. Metabolic rate for *P. cingulatus* levelled off at higher temperatures, however, suggesting that the thermal optimum for the species is approximately 21.5°C. This could be a major limiting factor for the species to meet its energetic requirements at higher stream temperatures in the system (see Figure 6b).

There were less consistent patterns in the allometric scaling of metabolism for both species, however, with no significant effect of body mass on respiration rate in *P. cingulatus*. The protective casing of *P. cingulatus* was not included in the estimation of body mass (Meyer, 1989), but this may have an influence on its metabolic rate (i.e., more energy expended in carrying a heavier case) and could be considered in future studies of allometric scaling in armoured organisms. The 95% CI of the allometric slope for respiration rate in *L. riparia* included the prediction of 0.67 from Euclidean geometric scaling, but not the prediction of 0.75 based on fractal networks (Savage et al., 2004). Thus, our results align with the general lack of consensus for a single universal allometric scaling exponent for respiration rate (Bokma, 2004; Isaac & Carbone, 2010; White, Cassey, & Blackburn, 2007), with suggestions that it may vary depending on environment and taxonomy (Ehnes, Rall, & Brose, 2011; Glazier, 2010).

### 4.3 Energetic efficiency and population abundance

The decline in energetic efficiency for both predators as stream temperature increased (Figure 6a,b) has previously been shown in terrestrial invertebrates (Rall et al., 2010; Vucic‐Pestic et al., 2011; but see Sentis et al., 2012). Such energetic constraints seem inevitable at higher temperatures unless organisms can sufficiently increase their food intake (Johansen et al., 2015), alter their feeding behaviour to target more energetically valuable resources (O'Gorman et al., 2016), or acclimate and even adapt to warmer conditions over time (Sentis et al., 2015). Even then, population persistence may be determined by top‐down control or the availability of sufficient resources at lower trophic levels (Johansen et al., 2015). Here, energetic constraints could have been offset by the greater availability of Simuliidae prey in the warmer streams at Hengill (Figure 3c), with energy intake exceeding expenditure for *L. riparia* at all stream temperatures (i.e., *y* > 1). Nevertheless, *P. cingulatus* was energetically inefficient (i.e., *y* < 1) above 17°C, potentially reflecting its much greater activation energy of metabolism (1.07 eV) relative to feeding rate (0.23 eV); that is, it does not sufficiently increase its feeding rate to meet its higher metabolic demands in warmer streams. Given the proximity of this species to its physiological and energetic thresholds (Figures 5c and 6b, respectively), energetic efficiency was a strong predictor of population abundance (Figure 6d). The occurrence of *P. cingulatus* at temperatures where *y* < 1 indicates that the species supplements its diet with other prey (Figure 2), particularly resources that require less energy to capture, such as organic vegetable matter (Otto, 1974).

Conversely, energetic efficiency was a poor predictor of population abundance in *L. riparia*, which had a surplus of energy across all temperatures (Figure 6c). This suggests factors other than energetic limitation determine population size in this species, for example habitat complexity or food web structure. The species prefers moss habitat (Wotton & Merritt, 1988), which is more abundant with increasing stream temperature at Hengill, and may support larger populations in warmer streams (Guðmundsdóttir et al., 2011). Additionally, we only considered one prey species in these experiments and, although Simuliidae are the major trophic pathway for *L. riparia* (see Figure 2), prey switching may help to meet its energy demands. While predation pressure from higher trophic level organisms was likely to be weak in our study streams (see Figure 2), we also cannot rule out the possibility that top‐down control from predators such as brown trout, or behavioural interactions with other organisms in the food web, could override the effects of energetics. Thus, relationships between energetics, temperature and population abundance may be complex and dependent on particular species traits, or traits of the food web in which they are embedded. Furthermore, behavioural mechanisms to offset elevated metabolic demands with warming must be considered. For example, a reduction in activity levels at higher temperatures in the wild may dampen predicted increases in metabolic rate, thus offsetting any potential shortfall in energy intake. Such behavioural responses should be taken into consideration for a more general application of our energetic efficiency framework for predicting abundance patterns.

Combined with the temperature dependence of attack rate, these results have potentially important implications for community structure and ecosystem functioning (Gilbert et al., 2014). Increased attack rates will strengthen short‐term interactions and could therefore destabilize population dynamics through increased predator feeding rates (Rall et al., 2010). Energetic inefficiency and eventual predator starvation will occur if metabolic demand outpaces resource intake, or the prey population cannot support predator feeding rates, destabilizing long‐term community dynamics due to the disproportionate loss of species from higher trophic levels (Petchey, McPhearson, Casey, & Morin, 1999). Associated release of lower trophic levels from top‐down control may alter primary production, food web stability and ecosystem functioning, while also reducing biodiversity (Fussmann et al., 2014; Kishi, Murakami, Nakano, & Maekawa, 2005). Alternatively, increased rates of prey growth and abundance could offset the negative effects of stronger interactions from predators (Berlow et al., 2009). Evidence for increased prey production due to faster growth and greater reproductive output in the warmer streams at Hengill (Hannesdóttir, Gíslason, Ólafsson, Ólafsson, & O’Gorman, 2013) indicates that prey populations could be sustained at higher temperatures in the long term, despite the stronger consumption pressure exerted on them.

## 5 CONCLUSIONS

We have demonstrated consistency in the temperature dependence of functional response parameters across field and laboratory settings, which supports their use in predictive modelling for estimating warming effects on natural systems (Binzer et al., 2016; Fussmann et al., 2014; Petchey et al., 2010; Vasseur & McCann, 2005). This consistency was found for two important invertebrate predators in freshwater streams that exhibit contrasting foraging modes and energetic efficiencies. This suggests that predator–prey interactions could respond to warming in broadly systematic and predictable ways, though the magnitude of the response depends on species identity and associated traits. Our results also suggest that estimates of energetic efficiency based on respirometry and functional response experiments offer a promising new way to identify consumer species that are on the threshold of energetic limitation and anticipate changes in their population abundances. This predictive ability will be crucial for successfully conserving and managing populations under accelerating rates of global warming. Our approach may also offer a move away from the current reliance on purely phenomenological climatic envelopes by incorporating the role of species interactions.

## AUTHORS' CONTRIBUTIONS

E.J.O.G., B.C.R., R.L.K. and G.W. conceived the study. L.C.A., E.H.S., B.G., R.L.K. and E.J.O.G. collected the data. B.C.R. developed the modelling approach. L.C.A., B.C.R., E.J.O.G., E.H.S. and M.J. analysed the data. All authors wrote the paper.

## Data Availability

Data available from the Dryad Digital Repository: https://doi:10.5061/dryad.tr4v447 (Archer et al., 2019).
