# Supplementary material for: Consistent temperature dependence of functional response parameters and their use in predicting population abundance
Source: J Anim Ecol. 2019 Aug 9;88(11):1670–83. doi: 10.1111/1365-2656.13060 (PMC6899737; doi:10.1111/1365-2656.13060)

Supporting Information:

Consistency in the temperature dependence of functional response parameters and their use in predicting population abundance

Louise C. Archer[1,2,†], Esra H. Sohlström[3,4,5†], Bruno Gallo[1], Malte Jochum[3,4,6,7], Guy Woodward[1], Rebecca L. Kordas[1], Björn C. Rall[4,5,*], Eoin J. O'Gorman[8*].

[1] *Department of Life Sciences, Imperial College London, Silwood Park Campus, Buckhurst Road, Ascot, Berkshire SL5 7PY, UK.*

[2] *School of Biological, Earth and Environmental Sciences, University College Cork, Distillery Fields, North Mall, Cork, Ireland.*

[3] *J.F. Blumenbach Institute of Zoology and Anthropology, University of Goettingen, Untere Karspuele 2, 37073 Goettingen, Germany.*

[4] *German Centre for Integrative Biodiversity Research (iDiv) Halle-Jena-Leipzig, Deutscher Platz 5, 04103 Leipzig, Germany.*

[5] *Institute of Biodiversity, Friedrich Schiller University Jena, Dornburger Str. 159, 07743 Jena, Germany.*

[6] *Institute of Plant Sciences, University of Bern, Altenbergrain 21, 3013 Bern, Switzerland.*

[7] *Institute of Biology, Leipzig University, Deutscher Platz 5e, 04103 Leipzig, Germany.*

[8] *School of Biological Sciences, University of Essex, Wivenhoe Park, Colchester, CO4 3SQ, UK*

[†] These authors contributed equally to this work

*** Correspondence to:** Eoin O'Gorman (e.ogorman@essex.ac.uk), Björn Rall (bjoern.rall@idiv.de)

Table S1. Optimum parameter values with associated standard errors (SE), *z*-values, and *p*-values from the most parsimonious model according to BIC describing the natural mortality of Simuliidae estimated from predator-free controls. Parameters correspond to those listed in Equation 2. Note that *i* represents Field 2015 data or Lab 2015 data, no natural mortality occurred in the Lab 2013 experiments, and the subscript *j* refers to either *L. riparia* or *P. cingulatus*. Two best-fitting models were found for the *L. riparia* combined control data (ΔBIC within 2 units) so the mortality rate which resulted in the more biologically realistic functional response model (Type II *versus* Type I) is presented here.

Predator	Parameter	Dataset	Estimate	SE	*z*-value	*p*-value
L. riparia	m_{ij0}	Field 2015	0.007	0.139	-36.14	< 0.001
	m_{ij0}	Lab 2015	0.009	0.150	-31.45	< 0.001
	E_{mij}	Field 2015	0.326	0.165	1.973	0.048
	E_{mij}	Lab 2015	0.920	0.193	4.762	< 0.001
P. cingulatus	m_{ij0}	Field 2015	0.006	0.100	-51.55	< 0.001
	m_{ij0}	Lab 2015	0.009	0.102	-46.53	< 0.001
	Em_{ij}	Field 2015	0.541	0.131	4.136	< 0.001
	Em_{ij}	Lab 2015	1.004	0.136	7.372	< 0.001

Table S2. Model fitting scheme for describing the functional response of *L. riparia* feeding on Simuliidae. Parameters correspond to those listed in Equation 5. Parameters without the subscripts *Lab2013*, *Lab2015*, or *Field2015* represent a shared parameter estimate for a given dataset, *i.e.* are independent of experimental setting. The difference in Bayesian Information Criterion (ΔBIC) relative to the most parsimonious model for the combined, Lab 2013, Lab 2015, and Field 2015 datasets (highlighted in bold) is also shown. Note that the type of functional response model was determined by changing the Hill exponent parameter in Equation 5: h_{ij} = 1 (and b_{ij} = 0) for Type I, h_{ij} = 1 for Type II, and h_{ij} = 2 for Type III models.

Model	c_0	$c_{0Field2015}$	$c_{0Lab2013}$	$c_{0Lab2015}$	E_c	$E_{cField2015}$	$E_{cLab2013}$	$E_{cLab2015}$	b_0	$b_{0Field2015}$	$b_{0Lab2013}$	$b_{0Lab2015}$	E_b	$E_{bField2015}$	$E_{bLab2013}$	$E_{bLab2015}$	ΔBIC
Type I		x	x	x		x	x	x									16.7
Type I		x	x	x	x												8.6
Type I		x	x	x													21.6
Type I	x				x												68.8
Type I	x																64.3
Type II		x	x	x		x	x	x		x	x	x		x	x	x	26.0
Type II		x	x	x	x					x	x	x		x	x	x	19.2
Type II		x	x	x						x	x	x		x	x	x	26.6
Type II		x	x	x		x	x	x		x	x	x	x				19.3
Type II		x	x	x	x					x	x	x	x				9.6
Type II		x	x	x						x	x	x	x				16.0
Type II		x	x	x		x	x	x		x	x	x					14.1
Type II		x	x	x	x					x	x	x					4.2
Type II		x	x	x						x	x	x					26.9
Type II		x	x	x		x	x	x	x				x				15.5
Type II		x	x	x	x				x				x				5.5
Type II		x	x	x					x				x				11.3
Type II		x	x	x		x	x	x	x								10.2
**Type II**		**x**	**x**	**x**	**x**				**x**								**0.0**
Type II		x	x	x					x								17.7
Type II	x				x					x	x	x		x	x	x	32.0
Type II	x									x	x	x		x	x	x	35.5
Type II	x				x					x	x	x	x				21.0
Type II	x									x	x	x	x				24.8
Type II	x				x					x	x	x					18.0
Type II	x									x	x	x					40.2
Type II	x				x				x				x				44.3
Type II	x								x				x				41.6
Type II	x				x				x								39.4
Type II	x								x								44.6
Type III		x	x	x		x	x	x		x	x	x		x	x	x	40.1

Type III		x	x	x	x					x	x	x		x	x	x	31.9
Type III		x	x	x						x	x	x		x	x	x	33.2
Type III		x	x	x		x	x	x		x	x	x	x				30.2
Type III		x	x	x	x					x	x	x	x				21.7
Type III		x	x	x						x	x	x	x				23.2
Type III		x	x	x		x	x	x		x	x	x					33.0
Type III		x	x	x	x					x	x	x					26.1
Type III		x	x	x						x	x	x					41.5
Type III		x	x	x		x	x	x	x				x				30.9
Type III		x	x	x	x				x				x				22.8
Type III		x	x	x					x				x				25.9
Type III		x	x	x		x	x	x	x								26.8
Type III		x	x	x	x				x								19.8
Type III		x	x	x					x								34.5
Type III	x				x					x	x	x		x	x	x	53.3
Type III	x									x	x	x		x	x	x	49.7
Type III	x				x					x	x	x	x				42.6
Type III	x									x	x	x	x				39.4
Type III	x				x					x	x	x					48.6
Type III	x									x	x	x					64.3
Type III	x				x				x				x				61.9
Type III	x								x				x				56.8
Type III	x				x				x								61.8
Type III	x								x								65.7

Table S3. Model fitting scheme for describing the functional response of *P. cingulatus* feeding on Simuliidae. Parameters correspond to those listed in Equation 5. Parameters without the subscripts *Lab2015* or *Field2015* represent a shared parameter estimate for a given dataset, *i.e.* are independent of experimental setting. The difference in Bayesian Information Criterion (ΔBIC) relative to the most parsimonious model for the combined Lab 2015 and Field 2015 datasets (highlighted in bold) is also shown. Note that the type of functional response model was determined by changing the Hill exponent parameter in Equation 5: h_{ij} = 1 (and b_{ij} = 0) for Type I, h_{ij} = 1 for Type II, and h_{ij} = 2 for Type III models.

Model	c_0	$c_{0Field2015}$	$c_{0Lab2015}$	E_c	$E_{cField2015}$	$E_{cLab2015}$	b_0	$b_{0Field2015}$	$b_{0Lab2015}$	E_b	$E_{bField2015}$	$E_{bLab2015}$	ΔBIC
Type I		x	x		x	x							32.3
Type I		x	x	x									27.4
Type I		x	x										38.4
Type I	x			x									268.1
Type I	x												326.0
Type II		x	x		x	x		x	x		x	x	15.0
Type II		x	x	x				x	x		x	x	10.8
Type II		x	x					x	x		x	x	8.8
Type II		x	x		x	x		x	x	x			9.9
Type II		x	x	x				x	x	x			6.1
Type II		x	x					x	x	x			3.9
Type II		x	x		x	x		x	x				4.8
Type II		x	x	x				x	x				1.0
Type II		x	x					x	x				7.1
Type II		x	x		x	x	x			x			8.0
Type II		x	x	x			x			x			3.6
Type II		x	x				x			x			0.3
Type II		x	x		x	x	x						3.9
**Type II**		**x**	**x**	**x**			**x**						0.0
Type II		x	x				x						5.8
Type II	x			x				x	x		x	x	44.4
Type II	x							x	x		x	x	57.0
Type II	x			x				x	x	x			39.3
Type II	x							x	x	x			53.4
Type II	x			x				x	x				35.4
Type II	x							x	x				48.4
Type II	x			x			x			x			181.6
Type II	x						x			x			185.4
Type II	x			x			x						180.2
Type II	x						x						207.0
Type III		x	x		x	x		x	x		x	x	99.7

Type III		x	x	x				x	x		x	x	94.5
Type III		x	x					x	x		x	x	90.3
Type III		x	x		x	x		x	x	x			95.1
Type III		x	x	x				x	x	x			90.6
Type III		x	x					x	x	x			86.0
Type III		x	x		x	x		x	x				93.4
Type III		x	x	x				x	x				89.2
Type III		x	x					x	x				89.5
Type III		x	x		x	x	x			x			103.6
Type III		x	x	x			x			x			99.0
Type III		x	x				x			x			93.8
Type III		x	x		x	x	x						109.8
Type III		x	x	x			x						105.1
Type III		x	x				x						103.5
Type III	x			x				x	x		x	x	117.6
Type III	x							x	x		x	x	123.5
Type III	x			x				x	x	x			114.9
Type III	x							x	x	x			120.9
Type III	x			x				x	x				109.9
Type III	x							x	x				121.7
Type III	x			x			x			x			211.1
Type III	x						x			x			213.5
Type III	x			x			x						214.1
Type III	x						x						233.7

Table S4. Comparison of linear regression models for determining the body mass and/or temperature dependence of respiration rate for *L. riparia* and *P. cingulatus*. The degrees of freedom (df) and difference in Bayesian Information Criterion (ΔBIC) relative to the most parsimonious model (highlighted in bold) are displayed.

Predator	Regression model	df	ΔBIC
L. riparia	mass * poly(temperature,2)	7	9.8
	mass + poly(temperature,2)	5	3.3
	poly(temperature,2)	4	22.6
	mass * temperature	5	2.2
	**mass + temperature**	**4**	**0**
	mass	3	19.3
	temperature	3	62.1
	intercept only	2	68.2
P. cingulatus	mass * poly(temperature,2)	7	10.9
	mass + poly(temperature,2)	5	3.8
	**poly(temperature,2)**	**4**	**0**
	mass * temperature	5	6.7
	mass + temperature	4	3.8
	mass	3	0.1
	temperature	3	15.0
	intercept only	2	13.8

Figure S1. Density dependent mortality of Simuliidae for *L. riparia* experiments conducted in (a-d) the laboratory in 2013, (e-h) the laboratory in 2015 and (i–m) the field in 2015. Prey mortality was measured across a gradient of increasing temperatures and mortality rates were generated using parameters estimated from the most parsimonious model according to BIC, which included a temperature dependent linear mortality rate with increasing prey density (Table S1). Circles and solid lines are prey mortality in experimental units that included one individual of *L. riparia*; crosses and dashed lines are prey mortality in predator-free controls. Note that there was no prey mortality in the Lab 2013 predator-free controls.

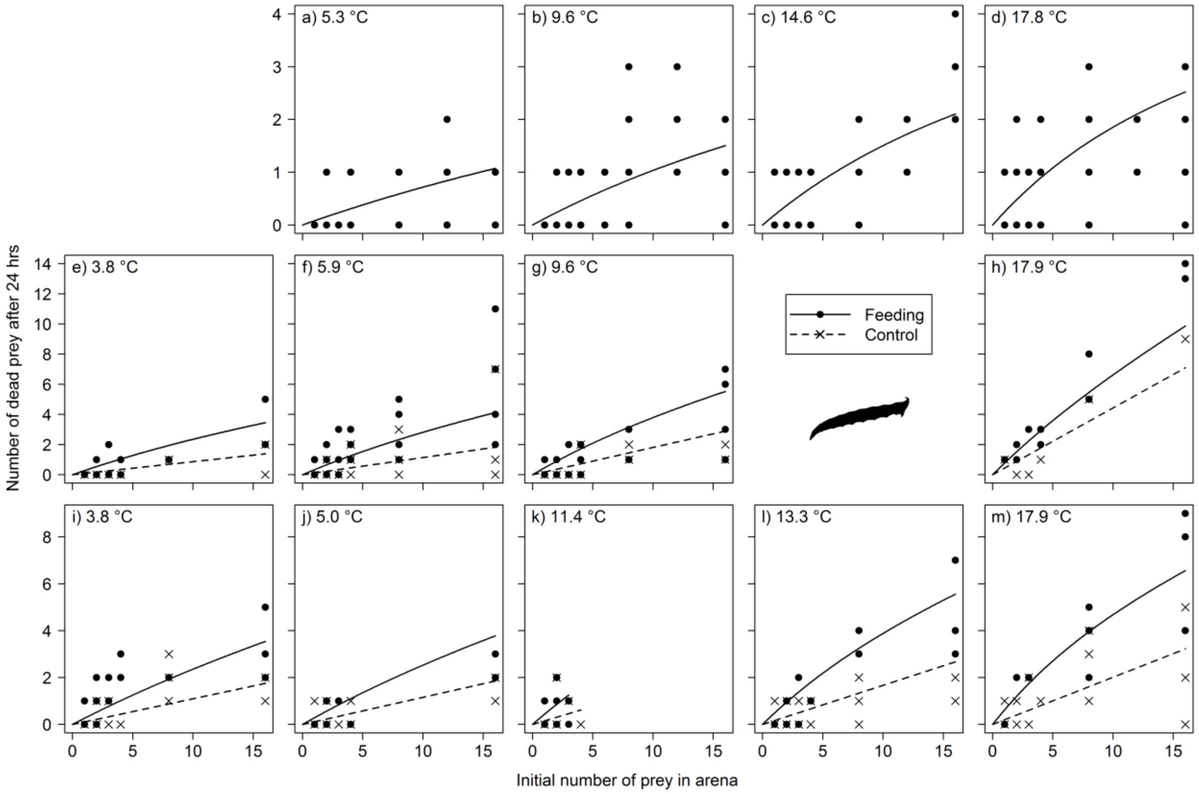

Figure S2. Density dependent mortality of Simuliidae for *P. cingulatus* experiments conducted in (a-d) the laboratory in 2015 and (e-i) the field in 2015. Prey mortality was measured across a gradient of increasing temperatures and mortality rates were generated using parameters estimated from the most parsimonious model according to BIC, which included a temperature dependent linear mortality rate with increasing prey density (Table S1). Circles and solid lines are prey mortality in experimental units that included one individual of *P. cingulatus*; crosses and dashed lines are prey mortality in predator-free controls.

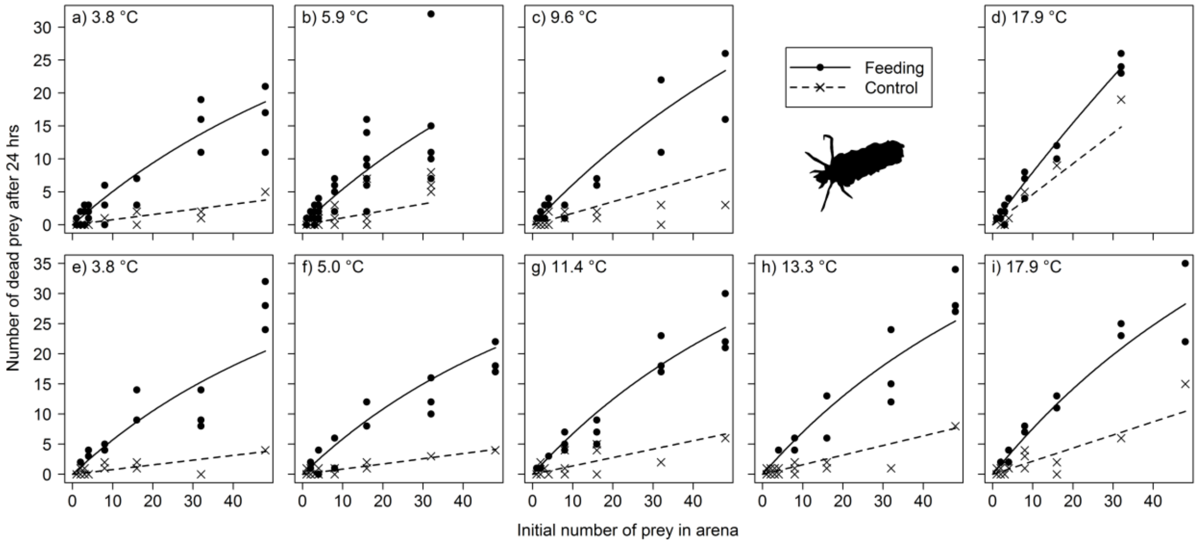

Figure S3. Length-weight relationship for the housefly larva, *Limnophora riparia*, calculated from data collected in the Hengill geothermal streams in June 2016. Linear regression: $y = 3.4032x - 3.2489$; $F_{1,65} = 198.3$; $p < 0.001$; $r^2 = 0.75$, where x is $\log_{10}$ body length (mm) and y is $\log_{10}$ body mass (dry weight in mg).

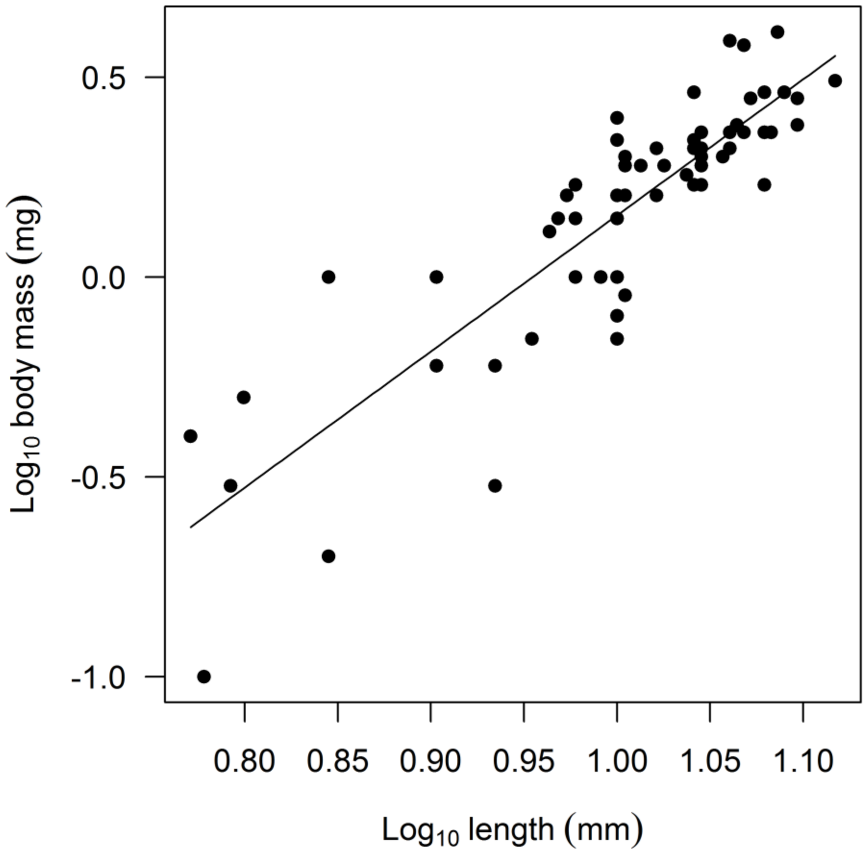

Figure S4. Feeding rate curves, for (a-c) *L. riparia* and (d-e) *P. cingulatus* feeding on Simuliidae, conducted across a gradient of temperatures in (a) the laboratory in 2013, (b,d) the laboratory in 2015, and (c,e) the field in 2015. Curves were generated using parameters in Table 1, estimated from the most parsimonious model according to BIC (Tables S2 and S3), after accounting for natural mortality of prey in the experiments (Figures S1 and S2).

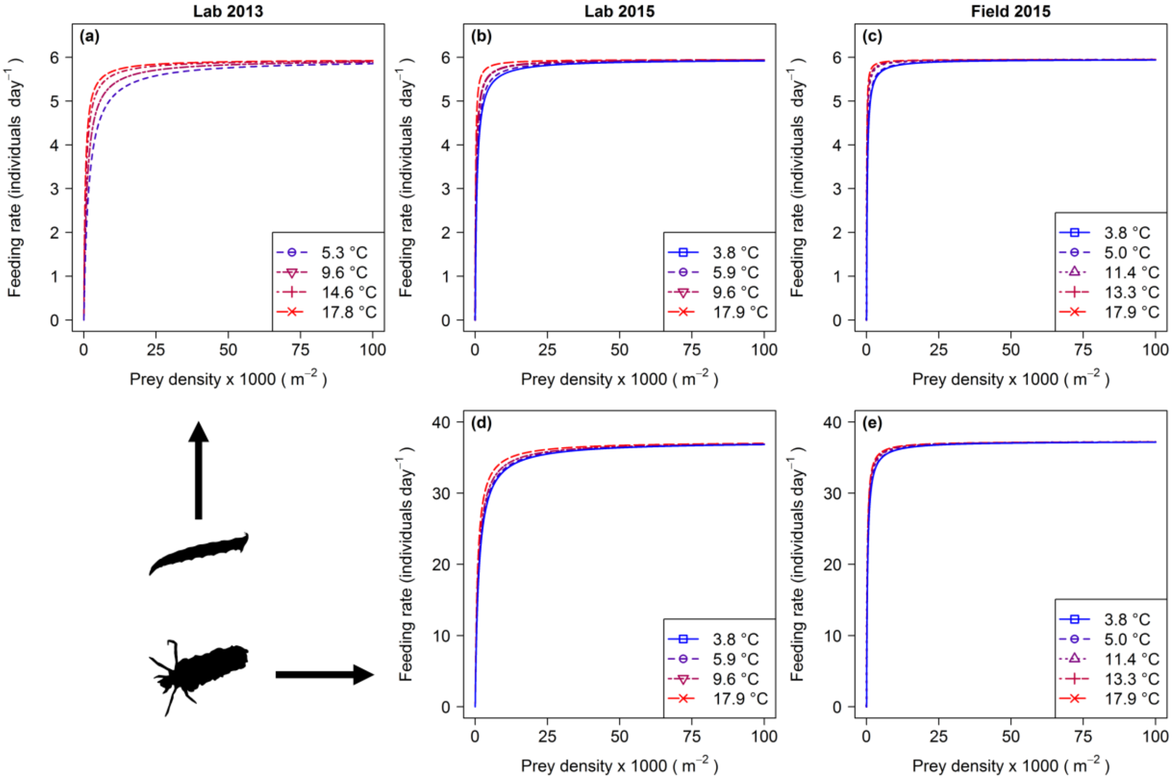

Supplement: Supplementary file 1 [file JANE-88-1670-s001.pdf]
